# Intestinal mucosal turnover in germ-free piglets infected with *E. coli*

**DOI:** 10.1007/s10735-024-10278-2

**Published:** 2024-12-04

**Authors:** Štefan Tóth, Zuzana Fagová, Monika Holodová, Kristína Čurgali, Eva Mechírová, Alexandra Kunová, Milan Maretta, Radomíra Nemcová, Soňa Gancarčíková, Marianna Danková

**Affiliations:** 1 Department of Histology and Embryology, Faculty of Medicine, Pavol Jozef Šafárik University in Košice, Šrobárova 2, 041 80 Košice, Slovak Republic; 2 Faculty of Medicine, Department of Neurology and L, Pasteur University Hospital, Pavol Jozef Šafárik University in Košice, Trieda SNP 1, 040 01 Košice, Slovak Republic; 3 Department of Microbiology and Immunology, University of Veterinary Medicine and Pharmacy in Košice, Komenského 73, 041 70 Košice, Slovak Republic; 4 Faculty of Medicine, Institute of Histology and Embryology, Comenius University in Bratislava, Sasinkova 4, 811 04 Bratislava, Slovak Republic

**Keywords:** Apoptosis, Turnover, Germ-free piglets, Escherichia coli, Intestine

## Abstract

We focused on investigation of *E. coli* infection influence on the turnover and apoptosis of intestinal mucosa. We have verified changes in proliferation and apoptosis in epithelial lining as well as in lamina propria of jejunum and colon of germ-free (GF) piglets as healthy control group and GF piglets in which at 5th day their gut was colonized with *E. coli* bacteria (ECK group). According to our results we detected significant increase in proliferation of the epithelial cells only in the jejunum of the ECK group, indicating a higher sensitivity to colonization with *E. coli*. Significant changes in the TUNEL assay and immunohistochemistry of other studied markers (TNF-α, Caspase-3 and HSP-70) were noted only in the lamina propria mucosae of both intestinal segments in the ECK group. In conclusion, we found that the commensal gut microbiota plays a role in regulation of the turnover rate in the epithelial lining, but also in the cells in the lamina propria mucosae in both intestinal segments, and that the host response is dependent on the colonising bacteria.

## Introduction

The gut microbiota plays a crucial role in maintaining metabolic homeostasis, as well as a myriad of physiological, neurological and immunological functions (Patil et al. 2020). In addition to being essential for nutrient digestion and absorption, the gut is the largest immune organ in the body. Activation of the gastrointestinal (GI) immune system leads to the production of a variety of specialised cells and signalling molecules, in particular pro-inflammatory cytokines such as tumour necrosis factor (TNF)-α, and results in intestinal mucosal injury and dysfunction, and consequently poor growth in pigs (Pié et al. 2004). A healthy GI tract is thought to be in a constant state of ‘controlled’ inflammation as a result of the proximity of a dense population of bacteria in the GI lumen (Liu 2015). Bacterial colonisation is a dynamic process, with the neonatal gut specifically tuned to promote tolerance to initially colonising bacteria (Hooper 2004). By detecting complex bacterial patterns, the gut senses the presence of luminal bacteria and distinguishes between pathogenic and commensal organisms, regulating tissue responses through a balance of inflammatory and apoptotic pathways (Kai-Larsen et al. 2007; Claud and Walket 2008; Conroy et al. 2009). The GI epithelium employs multiple innate defence mechanisms to combat microbial invaders, including epithelial integrity (Tóth et al. 2023), rapid epithelial cell turnover and innate immune responses (Kim et al. 2010). The interplay between bacteria, the intestinal epithelium and host innate responses is one of the most critical factors determining the fate of bacterial infections and disease outcomes.

The life cycle of intestinal epithelial cells is determined by the migration time from their site of origin at the base of the intestinal crypt to the apex of the intestinal villi, where they are shed into the lumen. A high rate of cell turnover in epithelium is distinctive feature of intestine, resulting in renewal of the entire intestinal epithelium every 3 to 5 days (Barker et al. 2008). This unique system is maintained by a balance between proliferation, differentiation and apoptosis of the intestinal epithelium. When this balance of cell death in the intestinal epithelium is disturbed, it leads to the development of several pathological conditions (Armacki et al. 2018).

Thus, in the intestinal epithelium, well-differentiated epithelial cells are constantly undergoing cell death, which contributes to physiological epithelial cell turnover and prevents persistent bacterial colonisation (Gunther et al. 2014). Therefore, an intensive, in-depth investigation of the role of cell turnover during invasion may help us to develop strategies against infection. In our previous experimental study, the influence of *E. coli* on the histomorphology and intestinal barrier of jejunum and colon was described in detail focusing on the cell populations such as goblet cells, enteroendocrine cells and mast cells (Tóth et al. 2023). The importance of understanding the processes in intestinal mucosa after bacterial colonisation is essential to reveal new strategies and defence mechanisms against diseases caused by microorganisms. Therefore, in this study, as the expansion of our previous work, we investigated the influence of *E. coli* bacterial colonisation on cell apoptosis and regeneration in the epithelial lining and in the lamina propria mucosa in the jejunum and colon of germ-free (GF) piglets. Our aim was also to evaluate changes in the inflammatory response of the jejunum and colon of GF piglets whose intestines were colonised with *E. coli* bacteria on day 5 after hysterotomy compared with GF piglets as healthy controls.

## Material and methods

The structure of the intestine is very similar in humans and piglets, including macroscopic features such as the ratio of intestinal length *per* kg bodyweight (Patterson et al. 2008). Therefore, the experiment was carried out on clinically healthy GF piglets (crossbred − Yorkshire × Pietrain). The piglets were obtained by the open hysterotomy method, kept in sterile isolators. The piglets were non-colostral and were fed autoclaved milk substitute (Sanolac Ferkel, Sano, Germany), diluted 1:5 with distilled water, sterilised by autoclaving at temperature 121 °C, pressure 1.3 MPa for 20 min. The milk substitute was fed to piglets individually from a glass bottle six times daily (2, 6, 10, 14, 18, 22 h), ad libitum. All piglets were fed every 4 h, i.e. 6 times daily. The piglets of healthy control group (HC, n = 4) were sacrificed using T61 (Intervet International B.V., Boxmeer, The Netherlands, dose: 0.3 ml/kg body weight) intracardially on 7th day. The animals in the infected experimental group (ECK, n = 5) were infected on the day 5 after birth by the dose 2 ml of prepared culture of *E. coli* O149: K88ac (1 × 104 CFU/ml). The *E. coli* O149: K88ac strain without enterotoxin production was obtained from the Danish Institute of Agricultural Science, Denmark. Overnight, culture of *E. coli* (1 ml) was inoculated into 50 ml Trypticase soy broth (Oxoid Unipath, Ltd., Basingstoke, UK) and cultivated at 37ºC in a water bath shaker (JULABO SW 2C, Labor Technic GMBH Selbach, Germany) for approximately 2 h to optical density 0.5 at 640 nm (corresponded to 1 × 108 CFU/ml). Subsequently, the bacterial culture was diluted in isotonic saline solution to obtain a final concentration of 1 × 104 CFU/ml. The purity of the broth culture was verified by spread plating on MC (MacConkey) agar and TSA (Tryptone Soy Agar) agar with sheep blood (Oxoid Unipath). Piglets were examined daily for clinical signs, including lethargy, pyrexia, diarrhoea and anorexia. The infected experimental piglets (ECK) were sacrificed using T61 (Intervet International B.V., Boxmeer, The Netherlands, dose: 0.3 ml/kg body weight) intracardially on 8th day. A routine microbiological control of gnotobiotic isolators was performed throughout the experiment. Microbiological swabs were taken from isolator walls, surface of animals and from their rectum. The samples were cultivated in PYG (Peptone Yeast Glucose) medium (Imuna, Slovak Republic). The microbiological control was verified every day on TSA agar with 5% ram’s blood (BBL, Microbiology systems, Cockeysville, USA). The presented experiment with protocol number 3629/05-221, was approved by the State Veterinary and Food Administration of the Slovak Republic. The animals were handled and sacrificed in a human manner in accordance with the guidelines established by the relevant Ethics Committee of the University of Veterinary Medicine and Pharmacy in Košice. Gastrointestinal (GI) tract was removed from the sacrificed piglets immediately. Segments of small intestine (jejunum) and large intestine (colon) as 1–2 cm long bioptic samples were obtained and washed with cold saline and fixed in 4% paraformaldehyde. After fixation, samples were embedded to paraffin. Histological sections 4–5 μm thick were cut from each paraffin block using a microtome (Leica RM2255, Germany).

### Goldner’s Masson trichrome staining

The sections of 4 µm thickness from gut were stained using Goldner-Masson trichrome assay (Goldner 1938). This staining method is capable of highlighting the fine structures of cells and tissues. Slides were mounted using a synthetic resin (Entellan; Merck, Germany). All stained sections were analysed histopathogically.

### TUNEL assay

Detection of apoptosis through the terminal deoxynucleotidyltransferase-mediated deoxyuridine triphosphate in situ nick end-labeling (TUNEL) in paraffin 4 µm thick sections was undertaken, using the TUNEL assay (DeadEnd TM Colorimetric TUNEL System, G7360, Promega; Madison, Wisconsin, USA) according to the manufacturer’s instructions. The nuclei were counterstained using Mayer’s haematoxylin (32750; SIGMA-ALDRICH, Co; St. Louis, MO, USA) and cover-slipped with Pertex (Histolab Products AB; Göteborg, Sweden).

### MCM2, TNF-α, caspase-3 and HSP-70 immunohistochemistry

Histological sections were deparaffinized and rehydrated. Endogenous peroxidase activity was blocked with 3% H_2_O_2_ with methanol. Pre-treatment was performed in a microwave oven at 600 W for 15 min in 0.01 M citrate buffer at pH 6.0. For immunohistochemical detection, the all sections were treated with primary antibodies. Primary antibodies were labelled using a two-stage indirect immunoperoxidase technique. Primary and secondary antibodies were applied at the appropriate titre (Table 1). Positive cells for colorimetric immunohistochemical analyses were visualized by diaminobenzidine (DAB; SIGMA-ALDRICH, Co), and the nuclei were counterstained using Mayer’s haematoxylin and cover-slipped with Pertex.

**Table 1:** Antibodies employed in this experimental study for immunohistochemistry

Primary and secondary antibodies	Antibody working dilution	Incubation parameters^a^	Source/code
Anti-MCM2 primary antibody	1:200	1 h, 37 °C	Abcam (ab108935)
Secondary antibody included in Ultravision LP Detection system HRP Polymer & DAB Plus Chromogen	RTU^b^	40 min, RT^a^	THERMO (TL-060-HD)
Anti-TNF-α primary antibody	1:100	1 h, 37 °C	Abcam (ab6671)
Secondary antibody included in Ultravision LP Detection system HRP Polymer & DAB Plus Chromogen	RTU^b^	40 min, RT^a^	THERMO (TL-060-HD)
Anti-Caspase3 primary antibody	1:200	1 h, 37 °C	Cell Signaling (96662S)
Secondary antibody included in Ultravision LP Detection system HRP Polymer & DAB Plus Chromogen	RTU^b^	40 min, RT^a^	THERMO (TL-060-HD
Anti-HSP70 primary antibody	1:100	1 h, 37 °C	Neo Markers (MS-482-PO)
Secondary antibody included in Ultravision LP Detection system HRP Polymer & DAB Plus Chromogen	RTU^b^	40 min, RT^a^	THERMO (TL-060-HD)

^a^RT: room temperature; ^b^RTU: the antibody is supplied ready-to-use by the manufacturer

### Confirmation of antibody specificity

In order to establish the specificity of the immunohistochemistry, a negative control test was carried out. Primary antibodies were omitted in all immunohistochemical methods for negative control test. The negative control test was conducted in all groups.

### Histomorphological evaluation

All measurements were performed in order to ensure objectivity in blind conditions, by two observers (unaware of the experimental groups) for all experimental groups and methods, carrying out the measures of control and experimental samples of each segment of the gut under the same conditions. For the qualitative/histomorphological (Goldner-Masson trichrome assay) and quantitative (TUNEL assay and immunohistochemistry) analyses, we used five sections from both gut segments in all animals’ groups. All measurements were done using magnification 400x. All tissue sections were analysed for the presence of histopathological lesions according to their extent and topographical localisation, with particular emphasis on individual mucosal compartments of both intestinal segments.

### TUNEL assay and immunohistochemical evaluation

Positivity was evaluated in 10 randomly selected intestinal wall areas *per* section in microscopic high-power fields for each colorimetric method (TUNEL, MCM2, TNF-α, active Caspase-3, HSP-70). In *lamina epithelialis mucosae* (LEM), 10 different lengths of epithelial lining were measured, and the number of positive cells was counted. Afterwards, the number of positive cells was calculated *per* 1 mm of the intestinal epithelium.

In *lamina propria mucosae* (LPM), 10 randomly selected areas of connective tissue were measured, and the number of positive cells was counted. Afterwards, the number of positive cells was calculated *per* 1 mm^2^ of the intestinal mucosa connective tissue.

### Statistical analysis

For histomorphological evaluation and colorimetric analyses (TUNEL/MCM2/TNF-α/active Caspase-3/HSP-70) light microscope OLYMPUS BX50 with a digital camera OLYMPUS SP350 (Olympus; Tokyo, Japan) and Quick PHOTO Industrial 2.3 image analyser software (Promicra; Prague, Czech Republic) were used. The statistical analysis was performed in GraphPad InStat ver. 3.10 for Windows (GraphPad Software Inc., San Diego, CA, USA).

Quantitative evaluation of studied markers is expressed as mean + S.E.M. (standard error of the mean). The significance of the differences between experimental groups was analysed using Kruskal–Wallis (Nonparametric ANOVA) and Dunn's post *hoc* Multiple Comparisons Test. The value of *p* < 0.05 was considered as statistically significant.

## Results

### Histomorphological analysis of jejunal and colonic mucosa

**Fig. 1:**
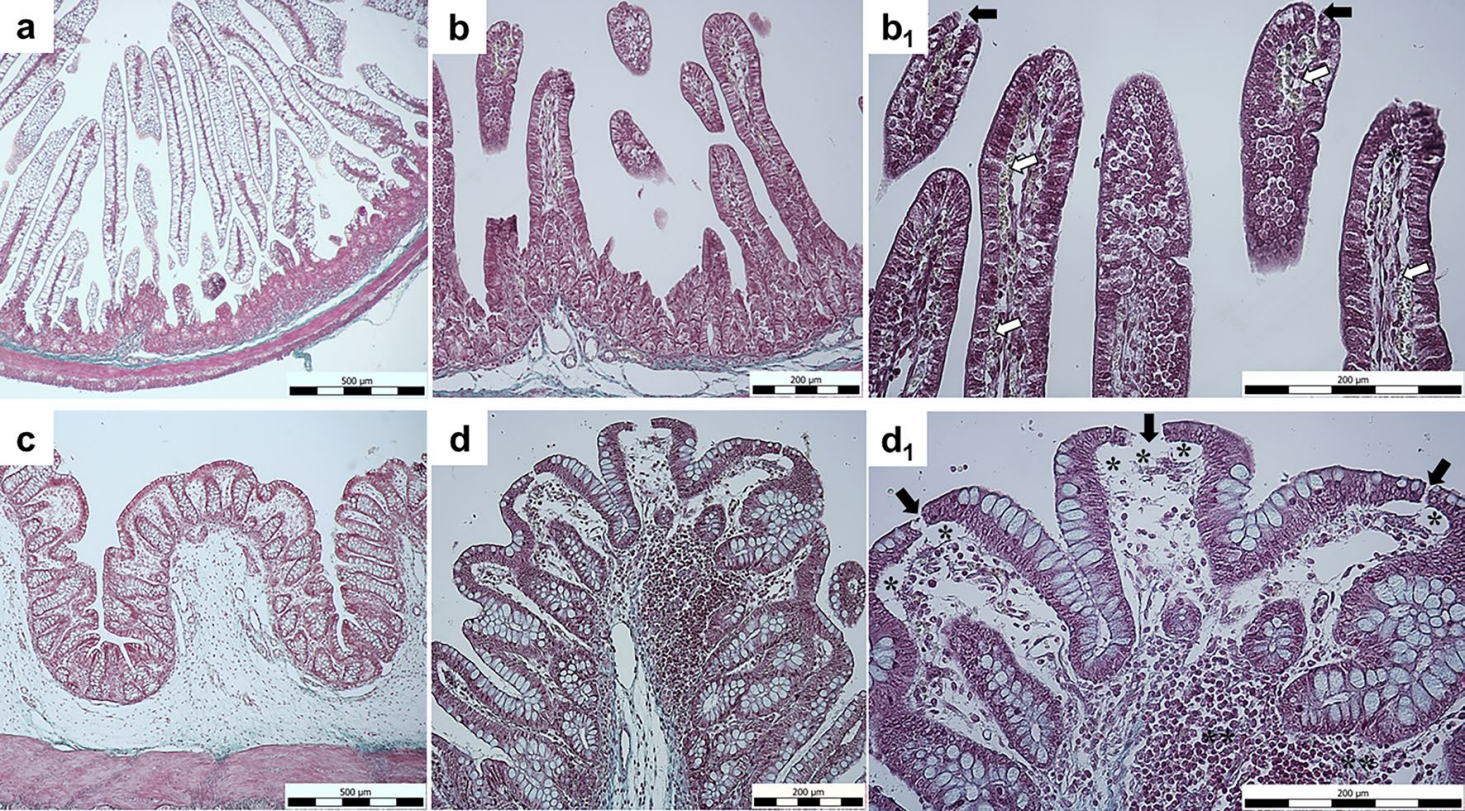
Histomorphological evaluation. **a**–**d** Representative micrographs of histopathological analysis of jejunal and colonic mucosa for each condition (HC: healthy control group; ECK: experimental group infected with *E. coli*). **a** HC jejunum with minimal or no injury; **b** ECK jejunum; **b**_**1**_ detail of ECK jejunal villi with moderate histopathological lesions such as prominent capillary congestion and dilatation (white arrow) and subepithelial oedema (black asterisk) at each intestinal villus tip; **c** HC group colon without injury; **d:** ECK colon; **d**_**1**_ detail of ECK colonic mucosa with histopathological lesions such as superficial epithelial disruption (thick black arrow), moderate subepithelial lifting with oedema (black asterisk) and leukocyte infiltration (double black asterisks) in the LPM of the colonic mucosa (**a**, **c** scale bar = 500 μm; **b**, **b**_**1**_, **d**, **d**_**1**_ scale bar = 200 μm)

We have previously reported detailed differences in intestinal morphology, including changes in crypt depth and villus height, following *E. coli* infection of germ-free piglet intestines (Tóth et al. 2023) as well as distribution of enteric nervous system structures (Danková et al. 2022). The use of Goldner’s Masson trichrome staining method allowed visualisation of individual histological layers in the intestinal wall of HC and ECK animals (Fig. 1a, c). The jejunal and colonic mucosa in the HC group appeared almost intact compared to the ECK group. In the experimental ECK groups, we reported lesions exclusively in the mucosa of both intestinal segments. Capillary congestion and dilatation with mild subepithelial oedema were observed at the villus tip of the jejunal mucosa in the ECK group (Fig. 1b, b1). Lesions in the colonic mucosa included occasional disruption of epithelial continuity, mild subepithelial oedema and the presence of leukocyte infiltration in the mucosal connective tissue (Fig. 1d, d1). The aforementioned lesions extended maximally to the *lamina muscularis mucosae* (LMM) and not to the deeper layer of the intestinal wall. The other histological layers of the intestinal wall of jejunum and colon were intact and showed no degree of damage.

### TUNEL assay

**Fig. 2:**
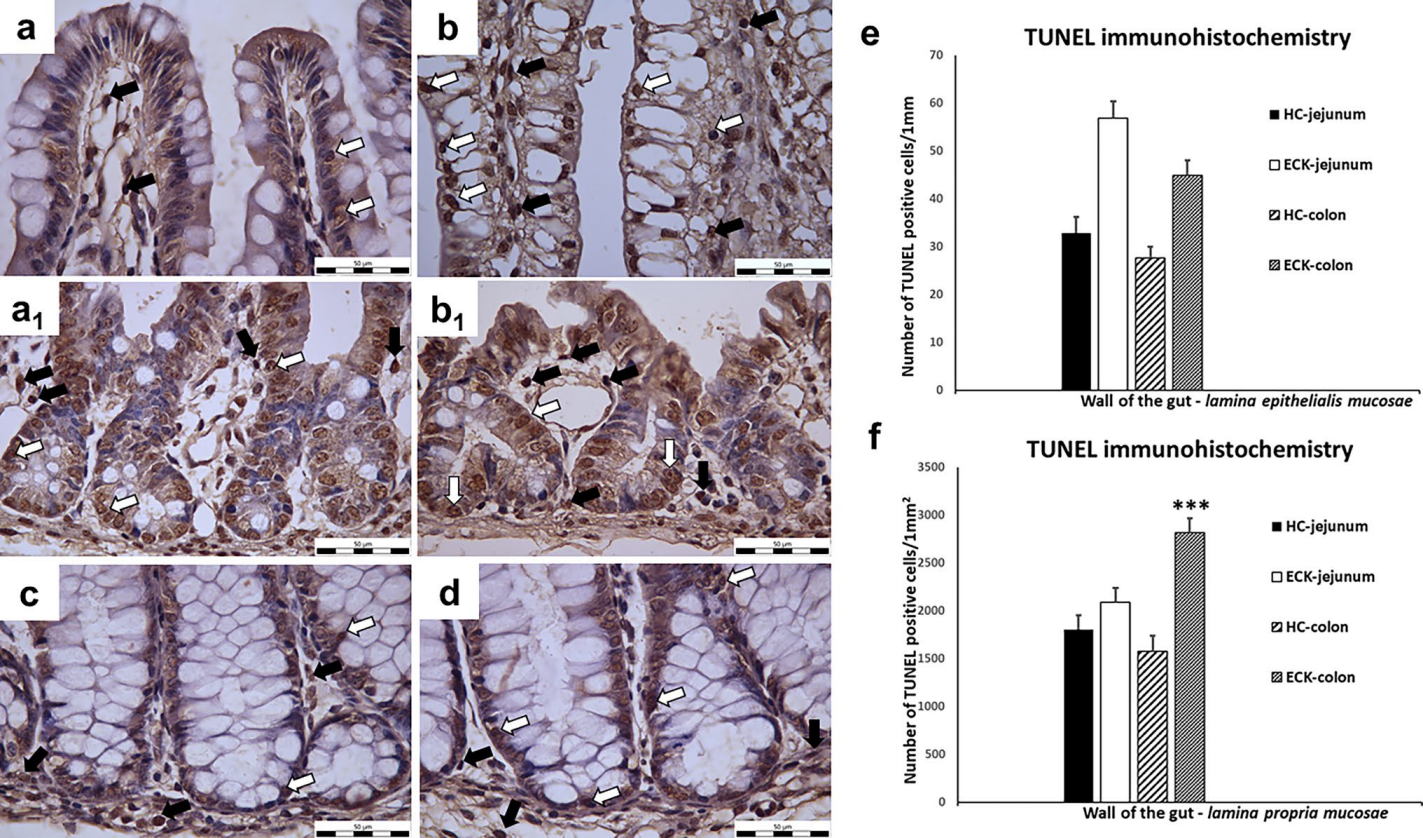
TUNEL assay—apoptosis in intestinal mucosa. **a**–**d** Representative microphotographs of immunohistochemical analysis of TUNEL-positive cells population in jejunal and colonic mucosa for each condition (HC: Healthy control group; ECK: Experimental group infected with *E. coli*). **a** jejunal villi of HC with minimal number of TUNEL-positive nuclei; **b** jejunal villi of ECK with increased number of TUNEL-positive apoptotic cells; **a**_**1**_ jejunal Lieberkühn crypts of HC with TUNEL-positivity; **b**_**1**_ jejunal Lieberkühn crypts of ECK with TUNEL-positivity; **c** colonic mucosa of HC with TUNEL-positive apoptotic cells in LEM and LPM around intestinal Lieberkühn crypts; **d** colonic mucosa of ECK with TUNEL-positivity (white arrows—TUNEL-positive nuclei of apoptotic cells in the LEM; black arrows—TUNEL-positive nuclei of apoptotic cells in the LPM; scale bar = 50 μm); **e**, **f** Graphs showing the TUNEL positivity in the intestine for each condition. **e** A non-significant increase in TUNEL positive cells in the LEM was observed in both intestinal segments of the ECK group compared to HC group. **f** The number of TUNEL positive cells in the LPM was significantly increased in the colon of ECK piglets compared to the colon of the control HC group (***indicates the values differ significantly from the colon of HC piglets at *p* < 0.001). Data are expressed as M + S.E.M

The TUNEL assay is based on the enzymatic ability of terminal deoxynucleotidyltransferase (TdT) to catalyze a template-independent addition of deoxyribonucleotide triphosphate to the 3′-OH ends of double or single-stranded DNA. The non-significant differences were found in the number of apoptotic cells detected by TUNEL assay in the LEM. A 42.8% increase in the number of TUNEL-positive cells was detected in the jejunal LEM of the ECK group compared to the HC group. A slight increase (40%) was also observed in the colonic LEM of the ECK group compared to the HC group. A non-significant increase (13%) was also detected in the jejunal LPM of the ECK group (13%) compared to the HC group. The results of the TUNEL assay showed the presence of a significantly (*p* < 0.001) higher number of apoptotic cells (43.95%) in the colonic LPM of the ECK group compared to the HC group (Fig. 2). The differences between apoptosis and proliferation are shown in Tables 2 and 3.

**Table 2:** The course of histological changes in the jejunal and colonic mucosa by using selected markers (HC: Healthy control group; *ECK* Experimental group infected with *E. coli*; **p < 0.01; ***p < 0.001; the tendency of change ↓—decrease, ↑—increase). MCM2: proliferation; TUNEL: apoptosis; TNF-α: cell death receptor; aC3: apoptosis; HSP-70: molecular chaperones with cytoprotective and immunomodulatory functions

Intestinal segment & compartment/Marker	TUNEL	MCM2	TNF-α	aC3	HSP-70
HC-jejunum-LEM	↓	↓	↓	↓	↓
ECK-jejunum-LEM	↑	↑***	↑	↑	↑
HC-jejunum-LPM	↓	↓	↓	↓	↓
ECK-jejunum-LPM	↑	↑	↑***	↑***	↑***
HC-colon-LEM	↓	↓	↓	↓	↓
ECK-colon-LEM	↑	↑	↑	↑	↑
HC-colon-LPM	↓	↓	↓	↓	↓
ECK-colon-LPM	↑***	↑**	↑***	↑***	↑***

**Table 3:** Comparison between cell proliferation (MCM2 marker) and apoptosis (TUNEL assay) in the LEM and LPM of both intestine segments (*HC* healthy control group; *ECK* experimental group infected with *E. coli*)

Group/antibody (jejunum)	Number of MCM2/TUNEL positive cells/1 mm of LEM (mean)	+ S.E.M	*p*
HC/MCM2	47	5.53	
HC/TUNEL	32	3.38	
ECK/MCM2	102	5.33	< 0.001: ECK/TUNEL
ECK/TUNEL	56	3.52	
Group/antibody (colon)	Number of MCM2/TUNEL positive cells /1 mm of LEM (mean)	+ S.E.M	*p*
HC/MCM2	89	5.307	< 0.001: HC/TUNEL
HC/TUNEL	27	2.372	
ECK/MCM2	96	5.239	< 0.001: ECK/TUNEL
ECK/TUNEL	44	3.009	
Group/antibody (jejunum)	Number of MCM2/TUNEL positive cells/1 mm^2^ of LPM (Mean)	+ S.E.M	*p*
HC/MCM2	3478	195.73	< 0.001: HC/TUNEL
HC/TUNEL	1806	148.78	
ECK/MCM2	3971	310.61	< 0.001: ECK/TUNEL
ECK/TUNEL	2087	148.84	
Group/antibody (colon)	Number of MCM2/TUNEL positive cells /1 mm^2^ of LPM (mean)	+ S.E.M	*p*
HC/MCM2	2959	237.73	< 0.001: HC/TUNEL
HC/TUNEL	1576	163.14	
ECK/MCM2	3672	210.25	< 0.05: ECK/TUNEL
ECK/TUNEL	2812	149.87	

### Immunohistochemical analysis of jejunal and colonic wall

#### MCM2 immunohistochemistry

The minichromosome maintenance family (MCMs) is a well-established group of proteins responsible for DNA synthesis (Blow 2005). Therefore, MCM2 as a marker of cell proliferation was used to identify proliferating cells in the intestinal mucosa. Significant changes in the number of MCM2-positive cells in the LEM of the jejunum of the ECK group were observed. There was a significant 53% increase (*p* < 0.001) in MCM2-positive cells in the jejunal LEM of the ECK group compared to the HC group, whereas only a non-significant 12% increase was observed in the colonic LEM.

**Fig. 3:**
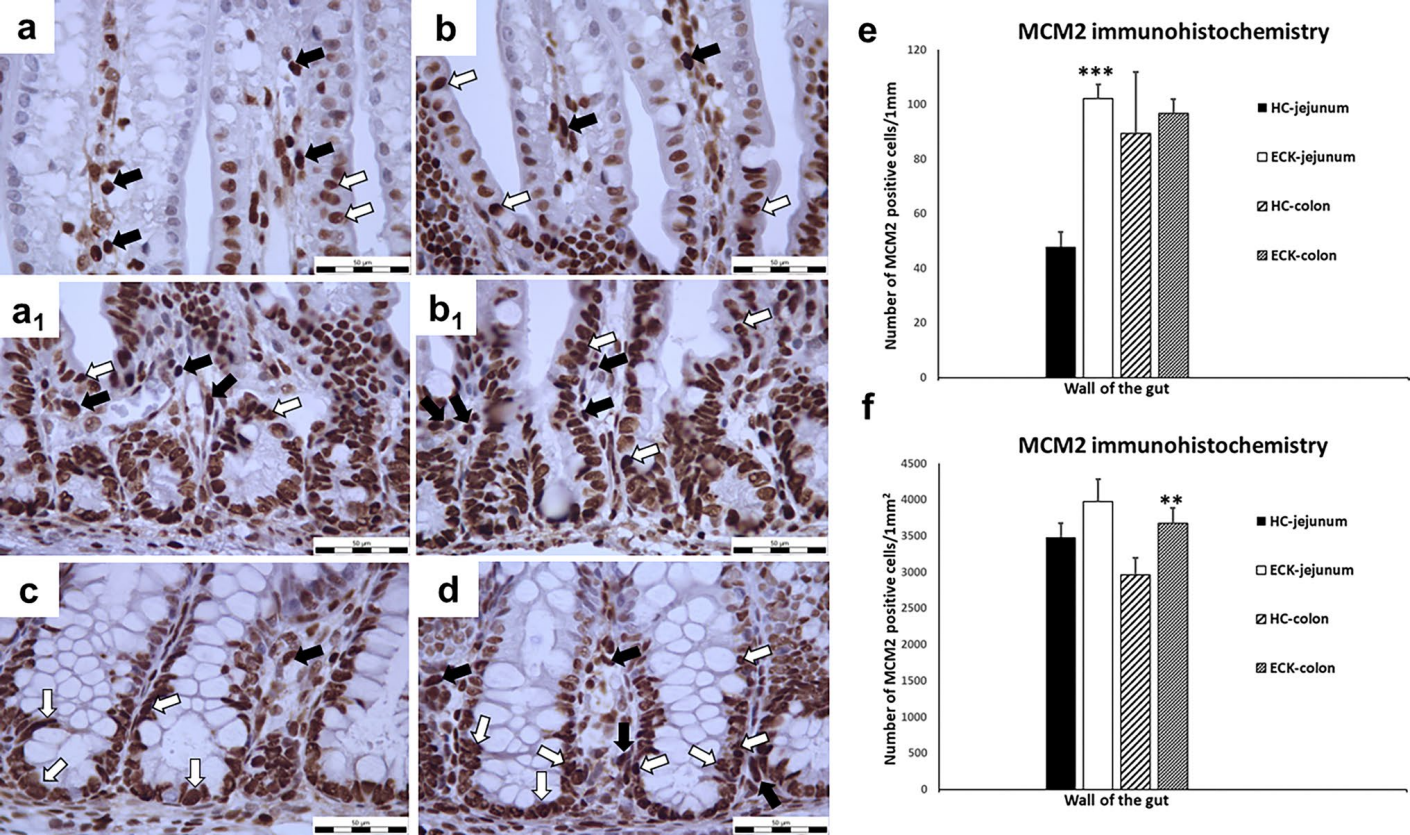
MCM2 immunohistochemistry—proliferation in intestinal mucosa **a**–**d** Representative microphotographs of immunohistochemical analysis of MCM2 in jejunal and colonic mucosa for each condition (HC: Healthy control group; ECK: Experimental group infected with *E. coli*). **a** jejunal villi of HC with lower number of positive nuclei for MCM2; **b** jejunal villi of ECK with increased number of MCM2-positive cells; **a**_**1**_ jejunal Lieberkühn crypts of HC with MCM2 nuclear positivity; **b**_**1**_ jejunal Lieberkühn crypts of ECK with MCM2 nuclear positivity; **c** colonic mucosa of HC with MCM2 positive nuclei in LEM and LPM around intestinal Lieberkühn crypts; **d** colonic mucosa of ECK with MCM2 positive nuclei (white arrows—nuclei of MCM2 positive cells in LEM; black arrows—nuclei of MCM2 positive cells in LPM; scale bar = 50 μm); **e**–**f** Graphs showing the MCM2 positivity in the intestine for each condition. **e** The number of MCM2 positive cells in the LEM was significantly increased in the jejunum of ECK piglets compared to the jejunum of the control HC group (***indicates the values differ significantly from jejunum of HC piglets at *p* < 0.001). **f** Significant increase in MCM2 in the LPM was observed in colon of ECK group compared to colon of HC group (** indicates the values differ significantly from colon of HC group at *p* < 0.01). Data are expressed as M + S.E.M

According to our histomorphological analysis, opposite results were found in the intestinal LPM (jejunum *vs.* colon). The experimental ECK group of GF piglets showed a more than twofold significant increase (*p* < 0.01) in the MCM2-positive cells (19.4%) in the colonic LPM compared to the HC group, while a non-significant increase of 7.5% was observed in the jejunal LPM (Fig. 3). The differences between apoptosis and proliferation are shown in Tables 2 and 3.

#### TNF-α immunohistochemistry

**Fig. 4:**
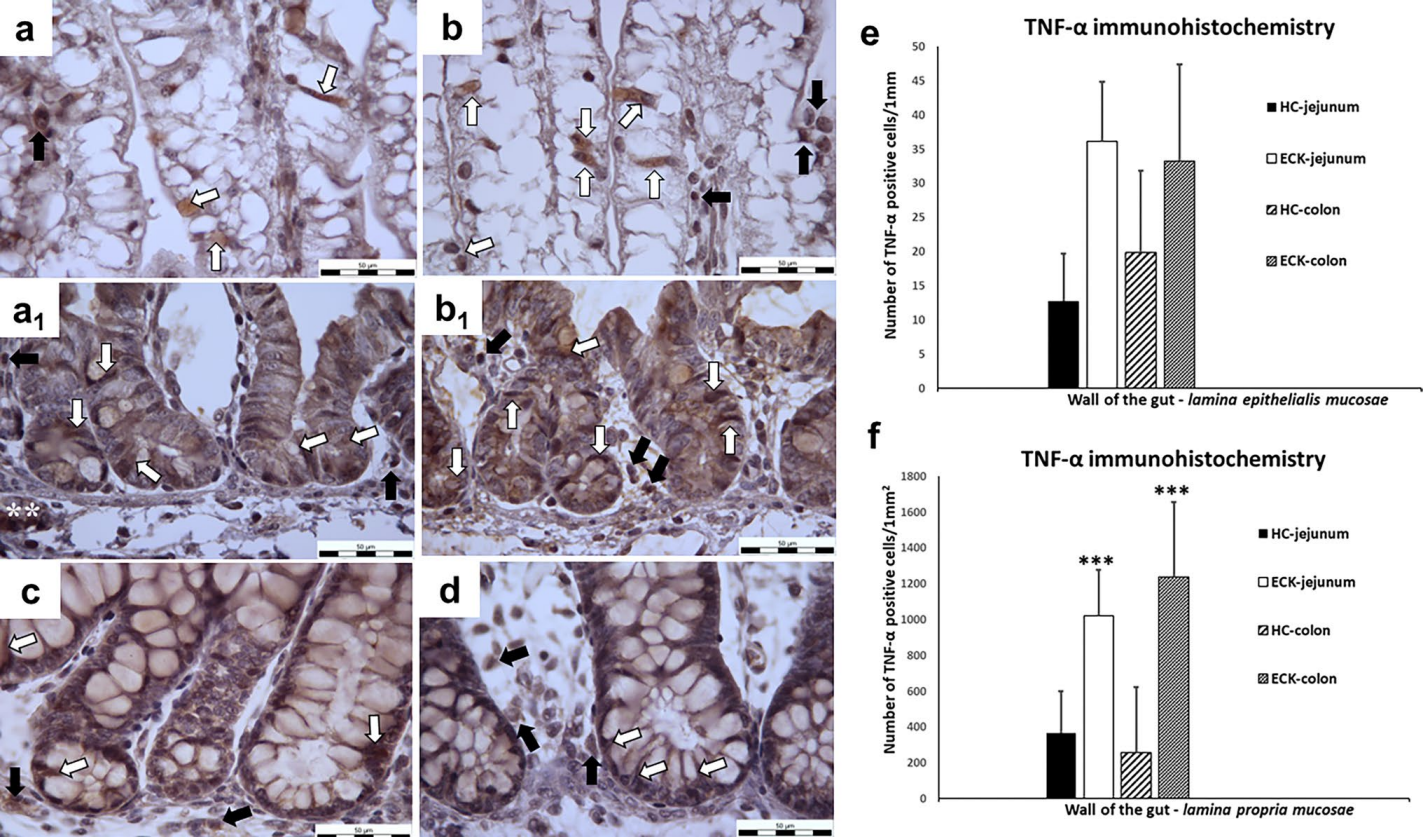
TNF-α immunohistochemistry **a**–**d** Representative microphotographs of immunohistochemical analysis of TNF-α positivity in the cells of the jejunal and colonic mucosa for each condition (HC: healthy control group; ECK: experimental group infected with *E. coli*). **a** jejunal villi of HC with minimal number of TNF-α positive cells; **b** jejunal villi of ECK with increased number of TNF-α positive cells; **a**_**1**_ jejunal Lieberkühn crypts of HC with TNF-α positive cells; **b**_**1**_ jejunal Lieberkühn crypts of ECK with TNF-α positivity; **c** colonic mucosa of HC with TNF-α cellular positivity in LEM and LPM around intestinal Lieberkühn crypts; **d** colonic mucosa of ECK with TNF-α positivity; (white arrows—TNF-α positive cells in the LEM; black arrows—TNF-α positive cells in the LPM; white double asterisk—positive neuroplasm of autonomic ganglionic cell population; scale bar = 50 μm); **e**, **f** Graphs showing the TNF-α positivity in the intestine for each condition. **e** A non-significant increase in TNF-α positive cells in the LEM was observed in the intestine of both segments of the ECK group compared to the HC group. **f** The number of TNF-α positive cells in the LPM was significantly increased in the both intestinal segments: jejunum of ECK piglets compared to the jejunum of the HC group and colon of ECK piglets compared to the colon of the control HC group (***indicates the values differ significantly from the jejunum of HC piglets at *p* < 0.001 and ***indicates the values differ significantly from the colon of HC piglets at *p* < 0.001). Data are expressed as M + S.E.M

TNF-α is a cytokine produced by immune, mesenchymal and epithelial cells. It is probably a critical mediator for many stimuli of pathological epithelial cell apoptosis, regulates the epithelial barrier in multiple ways, including mucus secretion, barrier permeability and cell proliferation (Leppkes et al. 2014; Parker et al. 2019). The previous observations and results prompted us to investigate whether *E. coli* infection also affected the production of TNF-α in the cells of the intestinal mucosa (Fig. 4). We observed a non-significant increase (66.37%) in the number of TNF-α positive cells in the jejunal LEM of the ECK group and a non-significant increase (40.23%) in the colonic LEM of the ECK group in comparison to the control HC groups. A similar trend was observed in the LPM of both segments. There was a significant increase (*p* < 0.001) in the number of TNF-α positive cells in the jejunum (64.22%) and colon (79.09%) of the ECK group compared to the control HC group. TNF-α positivity was predominantly observed in the cell cytoplasm in the intestinal mucosa of both segments. A very interesting immunohistochemical finding was the presence of TNF-α positivity in the cytoplasm of nerve cells in the autonomic ganglia near to the mucosa of both analyzed intestinal segments (Fig. 4a1).

#### Caspase-3 immunohistochemistry

**Fig. 5:**
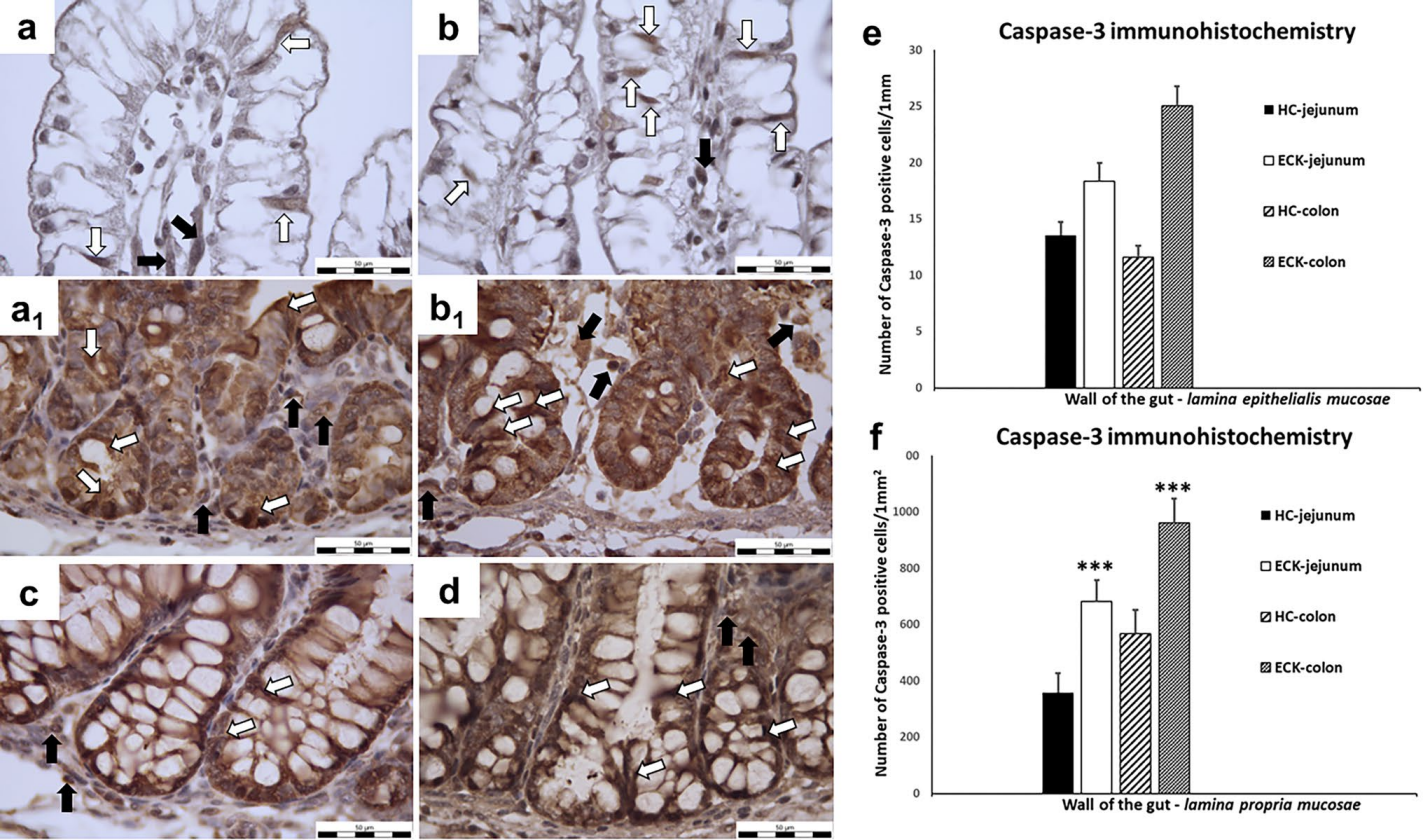
Active Caspase-3 immunohistochemistry. **a**–**d** Representative microphotographs of immunohistochemical analysis of active Caspase-3 (aC3) in jejunal and colonic mucosa for each condition (HC: healthy control group; ECK: experimental group infected with *E. coli*). **a** jejunal villi of HC with minimal number of positive aC3 cells; **b** jejunal villi of ECK with increased number of aC3 positive cells; **a**_**1**_ jejunal Lieberkühn crypts of HC with aC3 positive cells; **b**_**1**_ jejunal Lieberkühn crypts of ECK with aC3 positivity; **c** colonic mucosa of HC with aC3 positive cell cytoplasm in LEM and LPM around intestinal Lieberkühn crypts; **d** colonic mucosa of ECK with aC3 positivity; (white arrows—aC3 positive cells in the LEM; black arrows—aC3 positive cells in the LPM; scale bar = 50 μm); **e**, **f** Graphs showing the active Caspase-3 positivity in the intestine for each condition. **e** A non-significant increase in aC3 positive cells in the LEM was observed in both intestinal segments of the ECK group compared to HC group. **f** The number of aC3 positive cells in the LPM was significantly increased in the gut both segments: jejunum of ECK piglets compared to the jejunum of the control HC group and colon of ECK piglets compared to the colon of the control HC group (***indicates the values differ significantly from the jejunum of HC piglets at *p* < 0.001 and ***indicates the values differ significantly from the colon of HC piglets at *p* < 0.001). Data are expressed as M + S.E.M

Caspase-3 is a protein (also called CPP32 or apopain), encoded by the CASP3 gene. It is a member of the endoproteases (cysteine-aspartic proteases) family, which uses a cysteine, as the catalytic nucleophile, at its active site in order to cleave the target protein on its aspartic acid residue (Cade and Clark 2015). In the present study we observed changes in the number of active Caspase-3 (aC3) positive cells in the intestinal mucosa of the ECK groups. The results showed that the number of aC3 positive cells in the LEM was non-significantly increased in the jejunum (26.04%) and colon (53.67%) of the ECK groups compared to the HC group. Significant changes in the number of aC3 positive cells were observed in the LPM of the ECK groups in comparison to the control HC groups. The number of aC3 positive cells was significantly increased (*p* < 0.001) in both segments: jejunum (47.65%) and colon (40.08%) of the ECK groups compared to the HC groups (Fig. 5).

#### HSP-70 immunohistochemistry

**Fig. 6:**
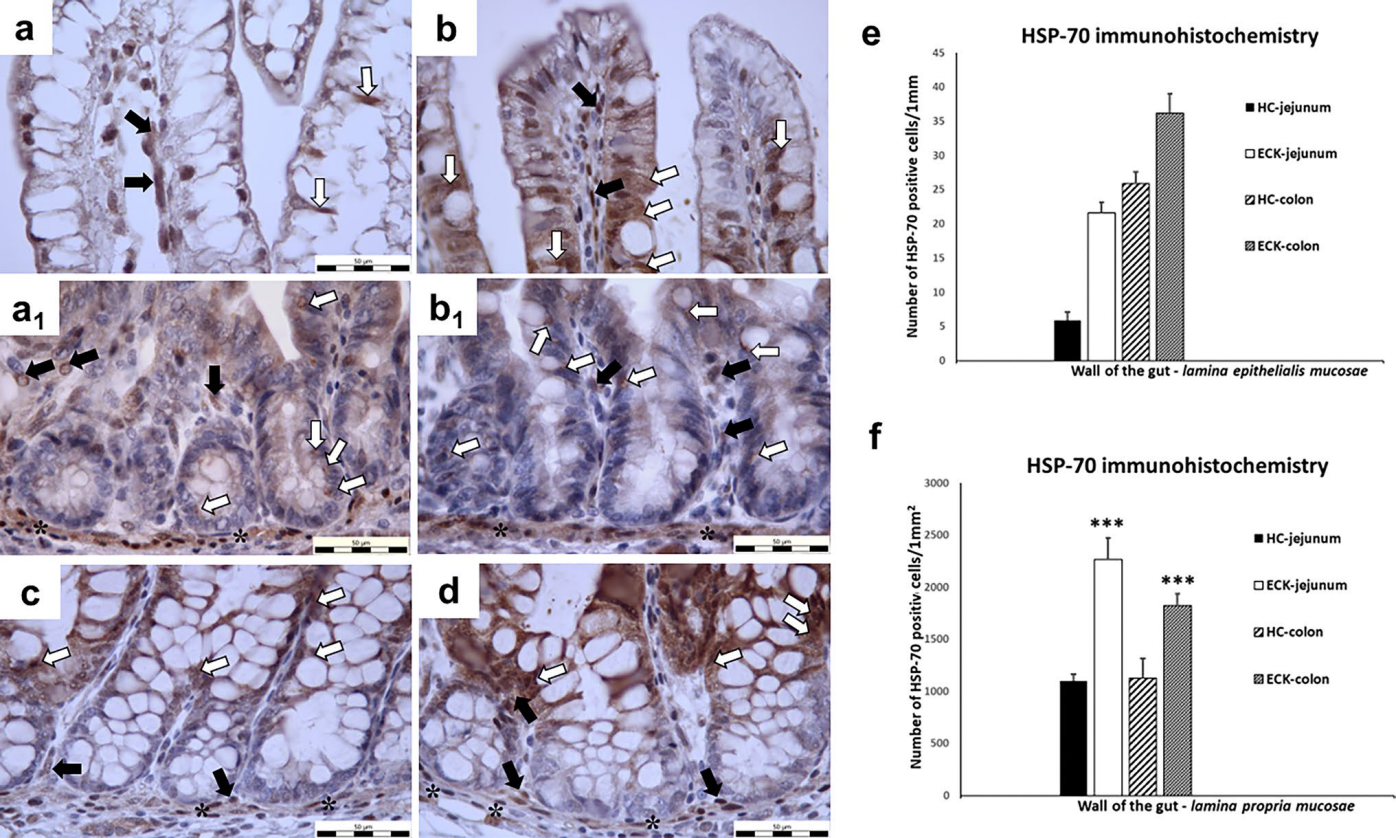
HSP-70 immunohistochemistry. **a**–**d** Representative microphotographs of immunohistochemical analysis of HSP-70 protein in jejunal and colonic mucosa for each condition (HC: healthy control group; ECK: experimental group infected with *E. coli*). **a** jejunal villi of HC with minimal number of HSP-70 positive cells; **b** jejunal villi of ECK with increased number of HSP-70 positive cells; **a**_**1**_ jejunal Lieberkühn crypts of HC with HSP-70 positive cells; **b**_**1**_ jejunal Lieberkühn crypts of ECK with HSP-70 positivity; **c** colonic mucosa of HC with HSP-70 positive cytoplasm of cells in LEM and LPM around intestinal Lieberkühn crypts; **d** colonic mucosa of ECK with HSP-70 positivity; (white arrows—HSP-70 positive cytoplasm of cells in the LEM; black arrows—HSP-70 positive cells in the LPM; asterisk—HSP-70 positive sarcoplasm of smooth muscle cells in the LMM; scale bar = 50 μm); **e**, **f** Graphs showing the HSP-70 positivity in the intestine for each condition. **e** A non-significant increase in HSP-70 positive cells in the LEM was observed in both intestinal segments of the ECK group compared to HC group. **f** The number of HSP-70 positive cells in the LPM was significantly increased in the gut both segments: jejunum of ECK piglets compared to the jejunum of the control HC group and colon of ECK piglets compared to the colon of the control HC group (***indicates the values differ significantly from the jejunum of HC piglets at *p* < 0.001 and ***indicates the values differ significantly from the colon of HC piglets at *p* < 0.001). Data are expressed as M + S.E.M

In the GIT mucosa, HSPs, in particular a 70-kDa heat shock protein 70 (HSP-70), are known to protect the GIT mucosa from toxic agents and ulcerogenic conditions (Nakamura et al. 1991; Watanabe et al. 2004). Therefore, we also focused on changes in the distribution of HSP-70 positive cells in the gut mucosa of GF piglets. HSP-70 positivity in the intestine of all experimental groups was detected predominantly in the cytoplasm of the cells (Fig. 6). The results showed that the number of HSP-70 positive cells in the LEM was non-significantly increased in the jejunum (72.76%) and colon (28.54%) of the ECK groups compared to the HC group. Significant changes were observed in the LPM of the ECK groups compared to control HC groups. The number of HSP-70 positive cells was significantly increased (*p* < 0.001) in both segments: jejunum (51.52%) and colon (38.17%) of the ECK groups compared to the HC groups. A very interesting finding was the presence of HSP-70 positivity in the sarcoplasm of the smooth muscle cells of the LMM near to the connective tissue of the mucosa of both analyzed intestinal segments (Fig. 6a1, b1, c, d). Based on the intensity of staining with DAB chromogen, it appears that more pronounced HSP-70 positivity was detected in the groups after infection with coliform bacteria (Fig. 6b1, d).

## Discussion

In our previous work, we described detailed morphological differences in intestines, including changes in crypt depth and villus height, after inoculation of GF piglets with *E. coli* (Tóth et al. 2023). It was revealed that epithelial barrier function and the mucosal immune system play important roles in protecting the intestinal tract of piglets from damage (Zhang et al. 2022; Tóth et al. 2023). In an attempt to identify other changes associated with bacterial colonisation, the actual study was mainly focused on changes in proliferation and apoptosis in the intestines of the GF piglets but also on TNF-α, aC3 and HSP70, which are in close relation with cell turnover.

The lifespan of epithelial cells in piglets’ gut is very short and therefore the dynamic balance between proliferation and apoptosis of intestinal epithelial cells is crucial for maintaining the integrity of the intestinal mucosa (Barker et al. 2008; Clevers 2013). We found that the commensal gut microbiota plays a role in regulating not only the epithelial turnover rate in the LEM, but also the cells in the LPM in both intestinal segments, and that the host response is dependent on the colonising organism.

In the present experimental model, we found an increase in the cell proliferation in the mucosa of both examined intestinal segments in the ECK group. On the other hand, despite the increase in proliferation, there was also a significant increase in the number of apoptotic cells, especially in the colonic mucosa of the ECK group. Apoptosis, this active and complex process occurs without inflammation or damage to surrounding tissues and requires energy input in the form of ATP—in contrast to necrosis (Kapezuk et al. 2020). In addition, the results led us to the interesting finding that if we observed a significant increase in proliferation in the LEM of the intestine in the infected ECK group then in the LPM we did not observe a significant increase in apoptosis. Conversely, if there was only a non-significant increase in cell proliferation in the LEM, then at the same time we observed a significant increase in apoptosis in the LPM of the ECK group. Significant increase in proliferation was noted only in the jejunal LEM of the ECK group. The colon of GF piglets is more resistant and less vulnerable to coliform bacterial infection in comparison to jejunum (Tóth et al. 2023). Apoptosis in response to bacterial infection may function to delete infected and damaged epithelial cells and restore epithelial cell growth regulation and epithelial integrity that are altered during the course of enteric infection (Kim et al. 1998). From the host point of view, the elimination of these populations of epithelial cells by apoptosis is very important and it could restore normal regulation of epithelial growth and differentiation, while maintaining the integrity of the epithelial mucosal barrier. The mechanisms responsible for microbially induced epithelial apoptosis leading to the increased intestinal permeability remain unclear.

Similarly to the mechanism of apoptosis itself, cytokines determine the proliferation and differentiation of intestinal epithelial cells. Cytokines of the transforming growth factor TGFα family are involved in cell proliferation and differentiation, while TNF-α and INF-γ cytokines determine apoptosis. When TNF-α binds to the tumor necrosis factor receptor 1 (TNFR1), it can act in higher parts of the villus to induce apoptosis, while its activity through the tumor necrosis factor receptor 2 (TNFR2) in the intestinal crypts can prevent excessive apoptosis and affect high levels of the p53 protein (Tartaglia et al. 1993; Mizoguchi et al. 2002; Williams et al. 2013; So and Ishii 2019). On the one hand, apoptosis has a protective function and on the other hand, parasitic micro-organisms use host apoptosis mechanisms to increase the permeability of the intestinal epithelium in order to cause infection. This explains why our results obtained for the distribution of TNF-α in the cells of the LPM of the ECK group were similar to those obtained by the TUNEL assay. In the LEM of the ECK group this significant similarity was not observed, invasion of the *E. coli* O149:K88ac strain without enterotoxin production in the ECK group had a similar non-significant effect as described by Jung et al. (1995) and Eckmann et al. (1993). The exaggerated apoptosis of epithelial cells in response to infection with pathogenic bacteria may be proposed by the host itself to eliminate infected and damaged epithelial cells. The execution of apoptosis is initiated by a pro-inflammatory programme and induced by either immunocompetent cells or epithelial cells. On the other hand, apoptosis could be a strategy of microbial pathogens to escape from the infected and exhausted host cell to invade deeper mucosal layers for a prolonged bacterial colonization (Hausmann 2010). The balance between apoptotic and anti-apoptotic signals triggered by TNF-α determines the accuracy in cell signalling (Cabal-Hierro and Lazo 2012). In summary, the pro-inflammatory cytokine TNF-α plays a fundamental role in maintaining intestinal homeostasis. It is thought to play a key role in both physiological and pathophysiological conditions in the gut. In any case, increased apoptosis in the intestinal epithelium is more likely to lead to intestinal inflammation (Nanci et al. 2007; Kajino-Sakamoto et al. 2008; Tatiya-Aphiradee et al. 2018), as evidenced by the results obtained in the LPM of the ECK group. TUNEL-positive apoptotic cells of the LPM in the ECK group were localised mainly in close vicinity of the intestinal epithelium basement membrane of both intestinal segments. Focusing on cell population in LPM, activated macrophages gathering in the superficial connective tissue of LPM (Iwanaga and Takahashi-Iwanaga 2022). Nagashima et al. (1996) demonstrated that lamina propria macrophages in the human large intestine vigorously phagocytosed epithelial cells, by a combined detection of epithelial cell-associated antigens and in situ end-labelling (TUNEL assay) in their cytoplasm.

Another key role in apoptosis is played by the caspase group of enzymes, which occur as inactive precursors of enzymes. After receiving the apoptotic signal, the cysteine protease is proteolyzed into an active form. Active caspase-3 (aC3) has been recognized as a critical mediator of apoptosis (Asadi et al. 2022). Caspase-3 plays an important role in the process of apoptosis as an effector caspase leading to the typical morphological and biochemical changes of the apoptotic cell, and therefore its activation is required in all apoptotic signalling pathways. In addition to apoptosis, caspase-3 is involved in the modulation of the inflammatory response as well as proliferation and cell differentiation processes, as shown by the results of Wang et al. (2023). In our study, we observed a non-significant increase in active caspase-3-positive cells in both segments of the LEM in the ECK group, in contrast to the LPM where the increase was significant. This observation demonstrated that apoptosis may occur as a primary event that enhances cell detachment, leading to cell loss. Bullen et al. (2006) found that cells always underwent apoptosis prior to desquamation and that apoptotic bodies were never found in the epithelial monolayer, which explains the presence of a significant increase not only caspase-3 positive cells but also TUNEL-positive cells in the LPM of the ECK group. Interestingly, Marchiando et al. (2011) used a broad-spectrum caspase inhibitor and showed that almost all shedding events were blocked, suggesting that caspase-3 cleavage is critical for cell shedding to occur. According to our results, we can conclude that both canonical pathways of apoptosis activation, the extrinsic and the intrinsic pathway, have been detected in the ECK intestinal mucosa. Although an increase of epithelial apoptosis in the ECK group is evident, its mechanisms and impact on epithelial barrier function have not been elucidated yet and was not taken under consideration of the LPM cell response (Butkevych et al. 2020).

Because caspases determine cell survival by promoting the cleavage of other proteins, their activity must be tightly regulated by factors that inhibit apoptosis, including heat shock proteins such as HSP-70. A point of no return is reached when the cell reaches a critical state of destruction, leading to cell death and the formation of structures called apoptotic bodies (Marchiando et al. 2011). Based on the results of the present study, we can conclude that *E. coli* infection led to a significant increase in HSP-70 positive cells in the intestinal mucosa of the ECK group. HSP-70 is an evolutionarily conserved protein whose expression increases the ability of the cell to survive several lethal conditions (Li et al. 2019). If epithelial cell apoptosis is not tightly regulated, this could lead to a barrier defect with subsequent microbial invasion and inflammation. The researcher Kazuhito Rokutan (2000) suggests that HSP-70 is one of the best-known endogenous factors that protect against cell and tissue injury under various pathological conditions, and chaperone inducers that selectively induce HSP-70 without toxic effects may be novel therapeutic approaches for the prevention and treatment of mucosal lesions. The important role of HSP-70 in the maintenance of barrier function was confirmed by Pearce et al. (2013), and an increase in HSP-70 production led to rescue inflammation in the intestinal mucosa (Rakoff-Nahoum et al. 2004). According to Zhong et al. (2010) immunolabeling showed that the HSP-70 protein was predominantly localized in the cytoplasm of epithelial cells. The signals of HSP-70 were distributed diffusely in the epithelial cells of the whole villi and intestinal gland rather than in the LPM and LMM. This histological finding is only partly consistent with the results of our immunohistochemical analysis. In our study, we observed the positivity for HSP-70 in LPM cells, and the finding of HSP-70 positivity in the sarcoplasm of smooth muscle cells of the LMM was particularly interesting. Such a finding of significant positivity in smooth muscle has not been described in the literature. As these findings might indicate that *E. coli* infection is a possible factor to enhance mucosal protective ability by inducing HSP-70 expression, we would propose the concept of “physiological adaptive cytoprotection” in the intestinal mucosa mediated by coliform bacteria infection. We suggest that modulation of HSP-70 production by microbial components may lead to an organised increase in HSP-70 expression in mucosal cells and is probably most important for improving gut protection. While the opposite is detrimental and may favour the development of chronic intestinal diseases (Arnal and Lalle’s 2016). This could be due to the heterogeneity of the cells in the LPM, and thus to different susceptibility and resistance to bacterial attack. The epithelial lining probably represents a more resistant compartment of the intestinal mucosa which, although sensitive to pathological conditions, has compensatory mechanisms that can buffer and correct the development of marked apoptotic changes. Future research should aim on better definition of microorganisms’ population and levels of microbial components for optimalization the expression of gut-protective HSPs and thus positively influence unwanted apoptosis. Moreover, this study may also open a new window for further research aimed on defined cell populations in the intestinal epithelium or lamina propria mucosae such as specific stem cells populations, but it also shows the potential for studying of different cell death pathways.

## Conclusion

The physiological role of apoptosis mechanisms in the intestine is one of the main functions of the epithelial barrier and thus an important aspect of mucosal protection. The intestinal epithelial cells are the first line of defence of the host cells against external factors including bacteria. Overall, the present data indicate that the intestinal epithelium does not respond to changes in the microbiota with a significant increase in apoptosis or cell proliferation, in contrast to cells in the LPM, where we observed significant changes in both segments of the intestine. Induction of these pathways results in activation of initiator caspases, leading to apoptosis commitment.

Infection with coliform bacteria induced significant changes in the LPM of jejunal and colonic mucosa. According to our analyses, *E. coli* infection had significant proliferative and proapoptotic effects. Based on the results and the comparison of both analysed intestinal segments and individual mucosal compartments, our analyses showed a marked cellular sensitivity in the LPM of colonic mucosa, where the changes were most pronounced in comparison to jejunum.

The results point to a complex interplay between microbiota, immunity and the intestinal barrier, which must work together to provide protection against pathogens while maintaining tolerance and balance.

Therefore, intensive research into the role of the cell balance between apoptosis and proliferation during bacterial invasion may help us to develop strategies against these infections in the future.

## Data Availability

No datasets were generated or analysed during the current study.
